# Suppressive effects of endophytic *Beauveria bassiana* on a stem boring pest of quinoa

**DOI:** 10.3389/ffunb.2026.1995993

**Published:** 2026-10-21

**Authors:** Neha Panwar, Jane E Stewart, Jorge R. Ibarra Caballero, Thomas Seth Davis, Adrianna Szczepaniec

**Affiliations:** 1 Department of Agricultural Biology, Colorado State University, Fort Collins, CO, United States; 2 Department of Forest and Rangeland Stewardship, Colorado State University, Fort Collins, CO, United States

**Keywords:** *Chenopodium quinoa*, entomopathogenic fungi, integrated pest management, microbial control, seed treatment

## Abstract

**Introduction:**

*Beauveria bassiana* is a widely used entomopathogenic fungus that can colonize plants endophytically, offering a potential strategy for managing endophagous insect pests that are difficult to target with conventional non-systemic insecticides.

**Methods:**

We evaluated whether *B. bassiana* can establish endophytically in quinoa, *Chenopodium quinoa* Willd. (Caryophyllales: Amaranthaceae) and suppress population growth of *Amauromyza karli* (Diptera: Agromyzidae), an emerging stem-boring pest in North American production systems. Quinoa seeds were inoculated by soaking in a conidial suspension prior to sowing.

**Results:**

Seed inoculation and the culture medium used to recover *B. bassiana* from surface sterilized plant tissues significantly affected colonization and detection of *B. bassiana*, respectively. The highest colonization frequency (70%) was detected at 7 days after germination using seed inoculation with *B. bassiana* + Silwet L-77 and Sabouraud dextrose agar (SDAY) with yeast extract as recovery medium. Colonization detection was time-specific and by 28 days after germination, *B. bassiana* was recovered only from plants in the B. bassiana + Silwet L-77 treatment with 10% colonization using SDAY as recovery medium. We further experimentally assessed the impact of *B. bassiana* inoculations on suppression of *A. karli* populations in potted quinoa plants exposed to natural field infestation over two years and in directly sown field plots. Seed inoculation did not significantly reduce larval density in either experiment, though, exit-hole density was significantly lower in B. bassiana-treated plants in the directly sown field experiment and showed a similar but non-significant trend in potted plants.

**Discussion:**

No mycosed larvae were recovered, suggesting that reduced exit-hole density might have resulted from sublethal or plant-mediated effects on larval development rather than direct fungal-induced mortality. These findings suggest that *B. bassiana* can establish endophytically in quinoa and indicate that seed treatments with *B. bassiana* may reduce infestation of *A. karli*, offering a potential strategy for managing this emerging pest.

## 1 Introduction

*Beauveria bassiana* (Bals.-Criv.) (Hypocreales: Cordycipitaceae) is a beneficial entomopathogenic fungus widely used as a biocontrol agent against a broad range of insects (Feng et al., 1994; Mascarin and Jaronski, 2016). Fungal entomopathogens are an effective alternative to synthetic pesticides and offer several advantages such as lack of toxic residues and lower risks to non-target organisms (Singh et al., 2017; Sharma et al., 2023). The fungus kills its host by penetrating the insect cuticle, then multiplying within the haemocoel of susceptible insects, where it produces toxic secondary metabolites such as beauvericin and depletes host nutrients, ultimately causing death (Vega et al., 2008). Under favorable environmental conditions, the fungus emerges from the insect cadaver and sporulates, enabling secondary transmission and self-perpetuation within the host population. In agricultural systems, this entomopathogen is mostly applied as an inundative spray directly to plants, soil, or target insects (Jaronski, 2010). However, the effectiveness of such applications depends on sufficient physical contact between viable conidia and the target insect (Ortiz-Urquiza and Keyhani, 2013; Sharma et al., 2023). Since *B. bassiana* depends on direct spore contact with the host cuticle, insects with concealed feeding habits (i.e., endophages) are difficult targets for externally applied inoculum (Tefera and Vidal, 2009; Singh et al., 2017). Pests that feed within plant tissue, soil, or other protected microhabitats such as stem borers, root-feeding larvae, or leaf miners are less likely to contact *Beauveria* spores deposited on the exposed plant surfaces, substantially reducing the likelihood of successful infection (Vega et al., 2008). Systemic insecticides, including neonicotinoid seed treatments, can suppress endophages as these chemicals are absorbed by plants and translocated through vascular tissues, allowing them to reach target insects (Dentzman et al., 2025). However, their use may be constrained by concerns regarding toxicity to pollinators and regulatory restrictions (Dentzman et al., 2025). One strategy to overcome this limitation is the facilitation of endophytic colonization in target plants.

*Beauveria bassiana* can live inside multiple host species as an endophyte without causing any apparent harm to the plant (Ownley et al., 2008; Panwar and Szczepaniec, 2024). Colonization can occur either naturally via penetration through stomatal openings and mechanical wounds, or it can be achieved artificially through targeted application methods such as seed treatments, soil drenching, or foliar sprays (Panwar and Szczepaniec, 2024). Once established *in planta*, the endophyte is well protected from unfavorable environmental conditions and substantially increases the possibility of contact with insects feeding internally on the colonized tissues (Ahsan et al., 2024). Beyond direct contact, endophytic entomopathogenic fungi can also improve plant defense responses and negatively impact herbivore performance by altering host nutritional quality or inducing sublethal effects such as reduced feeding and longer development period (Jaber and Araj, 2018; Russo et al., 2019; Panwar and Szczepaniec, 2024). However, the success of endophytic biological control may depend on host plant chemistry as secondary plant metabolites can inhibit *B. bassiana* growth and reduce virulence *in planta* (Mann and Davis, 2020).

Despite this limitation, the potential of endophytic *B. bassiana* to suppress internal feeders has been demonstrated in several crop systems. For instance, endophytic colonization by *B. bassiana* reduced feeding of *Iraella luteipes* (Thomson) (Hymenoptera: Cynipidae) by 70% in opium poppies, *Papaver somniferum* L. (Ranunculales: Papaveraceae) (Quesada-Moraga et al., 2009). Similarly, *Musa* spp. (Zingiberales: Musaceae) colonized by *B. bassiana* resulted in approximately 60% mortality of adult banana root borers, *Cosmopolites sordidus* (Germar) (Coleoptera: Curculionidae) (Akello et al., 2008). In tomato, *Solanum lycopersicum* L. (Solanales: Solanaceae), *B. bassiana* colonization reduced survival of *Tuta absoluta* (Meyrick) (Lepidoptera: Gelechiidae) larvae to zero within seven days of feeding on inoculated leaves as compared to 90% survival rate on untreated leaves (Silva et al., 2020). These collective findings illustrate the potential efficacy of this fungus against pests with concealed feeding habits and underscore the importance of expanding the use of endophytic *B. bassiana* in biological control.

This approach can be particularly useful for low-input agricultural systems such as quinoa, *Chenopodium quinoa* Willd. (Caryophyllales: Amaranthaceae). Quinoa has been cultivated in the United States since the 1980s, with total acreage scaling to approximately 3000 acres by 2021 (Szczepaniec and Alnajjar, 2023). Recently, infestations by the newly reported stem-boring fly, *Amauromyza karli* (Hendel) (Diptera: Agromyzidae), have caused substantial yield reductions, with losses reaching nearly 100% in some cases (Szczepaniec and Alnajjar, 2023). Adult activity peaks in quinoa fields in Colorado in late June, with larval infestations reaching over 90% by mid-July when the crop is in the early vegetative stage (Panwar et al., 2026). Larvae of *A. karli* feed within the quinoa stems, leading to the disruption of the vascular system and ultimately causing the stems to bend or fall down (lodging) and resulting in plant death. Since the initial detection of *A. karli* in 2021, North American growers have lacked effective management strategies to suppress this pest (Szczepaniec and Alnajjar, 2023).

Establishing *B. bassiana* as an endophyte in quinoa using seed treatment provides early-stage protection and offers a promising management tool because it may increase fungal exposure to the internally feeding larvae and affect larval development indirectly through endophyte-mediated plant defenses. To date, no endophytic entomopathogenic fungi have been evaluated against *A. karli*. However, *B. bassiana* has been found effective against other stem-boring agromyzids. For instance, Mutune et al. (2016) reported that seed inoculation with *B. bassiana* reduced feeding and oviposition punctures by bean fly, *Ophiomyia phaseoli* (Tyron) (Diptera: Agromyzidae) by nearly 70% and adult emergence by up to 83% as compared to untreated bean plants. Seed treatment represents a practical method for introducing entomopathogenic *B. bassiana* into the quinoa plant microbiome, as it can be easily integrated into existing planting operations and could potentially serve as an alternative to the use of neonicotinoid-treated seed. Despite this potential, the capacity of *B. bassiana* to successfully establish as an endophyte in quinoa following seed inoculations and its role in managing insect pests within this crop have not been evaluated.

The efficacy of seed inoculation may depend on the surfactant used in the fungal suspension. Surfactants influence liquid surface tension to improve conidial adhesion, wetting, and spreading on the seed coat. For instance, organosilicone surfactants such as Silwet L-77 can increase spreading in Lotus leaves (Tang et al., 2008). In addition, Silwet L-77 has been used to establish *B. bassiana* endophytically in crops such as pecan (Ramakuwela et al., 2020). To evaluate the success of these inoculations, establishment of fungal endophytes can be assessed by either culturing surface-sterilized plant tissues or detecting fungal DNA using molecular techniques such as qPCR (Dos Reis et al., 2022). Culture-based methods allow recovery of viable fungi, whereas DNA detection can indicate fungal presence without confirming viability. Because endophytic recovery may change as plants grow, sampling at multiple times after inoculation is useful to assess persistence.

Therefore, the objectives of this study were to determine whether *B. bassiana* can establish endophytically within quinoa and to assess the impact of endophytic colonization on suppressing *A. karli* infestation. To address these objectives, we first conducted a greenhouse experiment to identify the optimal surfactant and recovery medium for detection of endophytic establishment and then evaluated the efficacy of endophytic fungus against *A. karli* infestations in experiments using both potted and directly sown quinoa plants in the field. This is the first study to evaluate endophytic *B. bassiana* for the management of *A. karli* in quinoa. By addressing this gap, our research provides evidence for the potential of endophytic *B. bassiana* as a sustainable management tactic for *A. karli* in North American quinoa production systems and beyond.

## 2 Materials and methods

### 2.1 Fungal isolate and inoculum preparation

A commercial, registered *B. bassiana* strain GHA was obtained from the Davis laboratory at Colorado State University and maintained at 4°C until further use. This strain was previously characterized by Mann and Davis (2020) using ITS and EF1-α sequencing. To obtain fresh conidia for preparation of the liquid suspension used for seed soaking, the stored conidial powder was suspended in sterile distilled water, vortexed for three minutes to get a homogenous solution, and aliquots of the suspension were spread onto potato dextrose agar (PDA). The plates were incubated at 23 °C for approximately 14 days to obtain actively growing cultures.

To prepare the experimental inoculum, 10 mL of sterile distilled water was added to 14-day-old sporulating culture of *B. bassiana* grown on PDA and conidia were scraped gently from the surface of the medium using a sterile inoculating loop. The resulting suspension was filtered through a sterile cheesecloth to remove hyphae, and the filtrate was vortexed for three minutes to disperse the conidia and obtain a homogenous stock suspension. Conidial concentration of the stock suspension was estimated using a hemocytometer and adjusted to a final concentration of 1 x 10^7^ conidia/mL.

Conidial viability was assessed by plating 100 μL of the suspension on PDA medium. Plates were incubated for approximately 16 h at 23 °C. Three randomly selected groups of 100 conidia were observed under a microscope to determine percent germination. A conidium was considered germinated when the visible germ tube was longer than conidium diameter (Milner et al., 1991). Only suspensions with mean germination above 90% were used in the experiments.

### 2.2 Optimization of seed treatment and recovery medium to establish *B. bassiana* as an endophyte in quinoa

The experiment was conducted in a greenhouse in two blocks: the first one in June 2024 and the second in February 2025. The experiment was a complete randomized 3 x 2 factorial design with three levels of seed treatment (*B. bassiana* + Silwet L-77, *B. bassiana* + Tween 20 and sterile-water control) and two different recovery medium (PDA and Sabouraud dextrose agar supplemented with 1% yeast extract [SDAY]). Yeast extract was only added to the Sabouraud dextrose agar medium. The experimental unit was the individual potted plant, with 20 plants assigned to each of the six treatments within each block (N = 240).

Seeds of quinoa cultivar ‘Medano’ were obtained from Paul New, a grower in the San Luis Valley, Colorado, USA. Seeds received no pretreatments before the surface sterilization. Quinoa seeds were surface sterilized by immersion in 0.5% sodium hypochlorite for two minutes, followed by three rinses in sterile distilled water (Alquichire-Rojas et al., 2024). A 100 μL aliquot of the last rinse was plated on PDA to confirm the efficacy of surface sterilization. For each treatment, 20 surface-sterilized quinoa seeds were placed in a sterile petri dish and saturated with 5 mL of the assigned treatment solution. Seeds in the fungal treatments were soaked for 24 h in a conidial suspension containing 1 x 10^7^ *B. bassiana* conidia/mL (> 90% germination) and either 0.05% Silwet L-77 or 0.05% Tween 20. Seeds in the negative-control treatments were soaked for the same duration in sterile distilled water without surfactant. Aliquots of the conidial suspensions were also plated on the corresponding recovery media as a qualitative check for fungal viability.

Following treatment, seeds were dried on sterile paper towels and individually planted in 12.7-cm diameter pots in Lambert LM-40 high-porosity potting mix (Quebec, Canada). Plants were maintained under a 16–8 h (L:D) photoperiod with supplemental lighting cycle (430W High-Pressure Sodium + 65W LED), at day: night temperature of 23 C: 18 C and 70% relative humidity. Plants were kept randomly in mesh cages in the greenhouse at Colorado State University Plant Growth Facilities Insectary (Fort Collins, Colorado, USA). Plants were watered *ad libitum*, and no additional fertilizer was used during experiment. Endophytic colonization was evaluated at 7, 14, 21, and 28 days after germination, with five plants per treatment destructively sampled at each time point.

#### 2.2.1 Detection of endophytic colonization

To confirm the colonization of the endophyte-treated plants, different plant parts including leaves, stem, and roots were plated on the corresponding recovery medium. Quinoa plants were destructively sampled from each treatment on each sampling data and roots were gently rinsed with distilled water to remove adhering soil. Plants were then surface sterilized by immersion in 70% ethanol for 2 min, followed by 1% sodium hypochlorite for 3 min (Cunha et al., 2024). Plants were subsequently rinsed three times with sterile distilled water and allowed to dry on sterile paper towels. Using sterile scalpels, leaves were cut into approximately 1-cm^2^ sections, whereas stems and roots were cut into 1-cm-long segments. Five tissue pieces from each plant part were placed on the recovery medium assigned to that treatment and incubated at 25 ± 1 °C in the dark. To confirm the effectiveness of surface sterilization, 100 µL of water from the final rinse was plated separately on PDA plate. Tissue segments were examined daily for fungal growth. Re-isolated fungi were identified by comparing colony morphology and microscopic characteristics of the conidia with those of the original *B. bassiana* culture. A plant was considered colonized when *B. bassiana* was recovered from at least one of the tissue pieces from the sampled plant parts. Colonization frequency was determined by using the formula of Petrini and Fisher (1987) as modified by Posada et al. (2007):

colonization frequency = 100 × (number of plants from which fungal endophyte was re-isolated/total number of plants treated with).

Tissue specific recovery was also reported separately for roots, stems, and leaves. A plant was scored positive for a tissue when *B. bassiana* was recovered from at least one piece of the respective tissue. Tissue specific percentages were calculated with the same formula, with the numerator representing plants positive for that tissue.

### 2.3 Efficacy of *B. bassiana* against larval and exit hole densities of *A. karli* in potted quinoa plants

The efficacy of endophytic *B. bassiana* against *A. karli* was evaluated using potted quinoa plants during 2024 and 2025. The experiment was a completely randomized design with two treatments (*B. bassiana*- inoculated and control) randomly assigned to individual pots. Quinoa seeds assigned to the fungal treatment were inoculated with conidial suspension of *B. bassiana* using Silwet L-77 as described in Section 2.2, whereas control seeds were soaked in sterile distilled water. Plants were initially grown in 12.7 cm diameter pots in the Insectary greenhouse at Colorado State University Plant Growth Facilities.

In both years, potted plants were transferred to a commercial quinoa field in Mosca County in the San Luis Valley region of Colorado. The field was selected because consistently high levels of *A. karli* had been observed there in previous seasons (Panwar et al., 2026). Average canopy height of the plants already present in the field was greater than the potted plants. Before placement in the field, the base of each pot was removed so that the pots could be partially embedded in the field. This stabilized the pots under windy field conditions, facilitated plant establishment, and exposed plants to naturally occurring populations of *A. karli*.

In 2024, 64 pots were placed among four sections of the field, with each section containing eight *B. bassiana*-treated plants and eight control plants, to account for potential variation in naturally occurring *A. karli* pressure across the field. The distance between each section was approximately 20 feet. Pots were transferred to the field on 8 July 2024 and brought back to the laboratory at Colorado State University on 22 July 2024, resulting in a 14-day field exposure period. In 2025, the experiment was repeated with 40 pots distributed across five field sections with eight pots per section. Each section contained four plants treated with *B. bassiana* and four untreated plants. Pots were placed in the field on 15 July 2025. Plants were transferred to the field later in 2025 because germination and plant establishment occurred later than in 2024. Because adult A. *karli* activity typically declines to undetectable levels by late July, pots were removed from the field on 23 July resulting in a shorter exposure period compared to 2024. Before transportation, each bottomless pot was placed inside an intact pot to provide support during transport and subsequent handling.

After removal from the field, plants were maintained in the greenhouse for an additional two weeks to allow the development of *A. karli* larvae within the stems. At the end of the experimental period, all plants were destructively sampled to assess *A. karli* infestation. Plants that died in the field were not included in the infestation assessment. Plant mortality was associated with stem breakage caused by windy conditions in the field and was not associated with the treatment effect. In 2024, 15 of 64 plants died in the field (7 control plants and 8 *B. bassiana*-treated plants), whereas in 2025, 18 of 40 plants died (8 control and 10 *B. bassiana*-treated plants). Due to the limited number of plants available in this experiment, endophytic colonization was not assessed. Since endophytic establishment and persistence were not confirmed, this experiment only evaluated the impact of seed treatment with *B. bassiana +* Silwet L-77 rather than effect of endophytic colonization on *A. karli* densities.

Infestation by *A. karli* was assessed by counting the number of larvae present inside quinoa stems and exit holes on the stem surface (**Figure 1**). Exit holes are produced when mature larvae leave the stem to pupate in the soil and therefore serve as an indicator of larvae that had completed their development.

**Figure 1:**
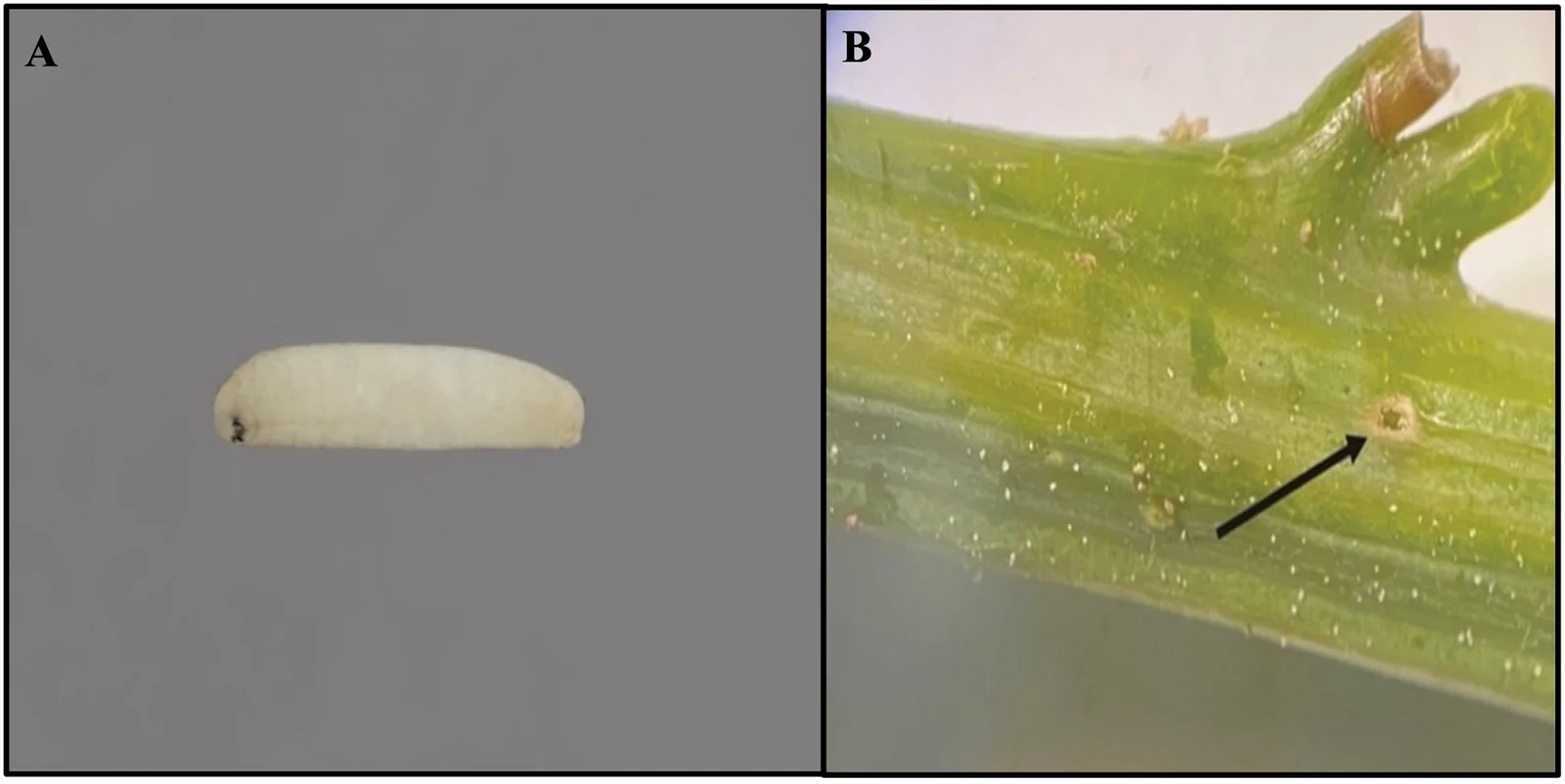
Representative images illustrating the larva of *Amauromyza karli* **(A)**, and exit hole on the stem (arrow) produced by an *A. karli* larva **(B)**. Reproduced from Szczepaniec and Alnajjar (2023) under the Creative Commons Attribution-NonCommercial (CC BY-NC 4.0) license.

Stems were split longitudinally using a sterile scalpel, and the number of larvae (both alive and dead; only one dead larva was found across the study and was included in the counts) and exit holes were recorded. Stem length was also recorded, and stem diameter was measured using a Vernier caliper. Brances were not included in the measurements. Stem volume was calculated as π(d/2)^2^h, where *d* is the stem diameter and *h* is the stem length. Larval and exit hole densities were expressed as number per cm^3^ of stem tissue. Plants with at least one larva or exit hole were classified as infested. Infestation incidence was calculated by dividing the number of infested plants by the total number of plants.

### 2.4 Efficacy of *B. bassiana* against larval and exit hole densities of *A. karli* in directly sown quinoa in field

The efficacy of quinoa plants grown from *B. bassiana*-treated seeds was evaluated under field conditions in 2026. The experiment was conducted as a randomized complete block design with two treatments, quinoa seed inoculated with *B. bassiana* using the procedure described in Section 2.2 and non-treated control seeds. The experimental unit was the individual plot, with six replicate plots per treatment (N = 12). Each plot contained six rows, each 20 ft long, and adjacent plots were separated by a 2 ft wide space.

The experiment was conducted in a commercial grower field in the San Luis Valley region of Colorado. Seeds were sown by hand on 26 May 2026 and covered with soil using a hand-push garden seeder. Plants were sampled on two sampling dates, i.e., 9 June 2026 and 30 June 2026. At each sampling date, 20 plants were randomly sampled from the middle rows of each plot (excluding outer border and corner rows). Sampled plants were uprooted from the soil to retain the roots and transported to the laboratory at Colorado State University on ice. Of the 20 plants collected from each plot, a subset of five plants was randomly selected to assess endophytic colonization using SDAY as the recovery media (Section 2.2.1), and the remaining 15 plants were used to assess infestation by *A. karli* (Section 2.3).

### 2.5 Statistical analyses

All statistical analyses were conducted in R (R Core Team, 2025). For the greenhouse experiment, endophytic colonization was analyzed using Firth’s bias-reduced logistic regression using *brglm2* package (v.1.1.0) to account for complete separation resulting from zero colonization in control plants (Firth, 1993; Kosmidis, 2025). A generalized linear model with a binomial error distribution was used to analyze the effects of surfactant, recovery medium, and time on the probability of endophyte colonization. The initial model included block, surfactant, recovery medium, sampling time, and all their interactions. Since none of the interactions were significant, the final model included only the additive effects. *Post hoc* comparisons were conducted using estimated marginal means with Tukey adjustment for multiple comparisons using the emmeans package (Lenth, 2025).

For the potted-plant field experiment evaluating suppression of *A. karli* (Section 2.3), data from 2024 and 2025 was analyzed separately. Within each year, the probability of plant infestation was analyzed using a generalized linear model with a binomial distribution, with treatment and section ID included as fixed effects. Larval and exit-hole counts were analyzed using generalized linear models with log links, and plant volume was included as an offset to account for differences in plant size. Count distributions were selected based on assessment of dispersion relative to a Poisson model. In 2024, larval counts were analyzed using a quasi-Poisson distribution because the data were overdispersed. Larval occurrence was extremely low in 2025, and no exit holes were observed in 2024, therefore no statistical analyses were conducted for these responses. In 2025, exit-hole counts were analyzed using a Poisson distribution because the dispersion parameter was approximately 1. Treatment and block were included as fixed effects in all generalized linear models.

For the direct-sown field experiment evaluating the efficacy of *B. bassiana* against *A. karli* (Section 2.4), data were analyzed using the plants collected on June 20 only. Plants sampled on June 9 were small and had no larvae or exit holes, so they were excluded from the analysis. Larval density, exit-hole density, stem length, stem diameter, and calculated stem volumes were analyzed separately using linear models with treatment and block as fixed effects. Model residuals were examined for normality and homogeneity of variance using diagnostic plots. Statistical significance was assessed at α = 0.05.

## 3 Results

### 3.1 Endophytic colonization in greenhouse experiment

The probability of recovering *B. bassiana* from surface-sterilized quinoa tissues varied with seed treatment (χ^2^ = 15.17, df = 2, P < 0.001), recovery medium (χ^2^ = 5.68, df = 1, P = 0.017), and sampling time (χ^2^ = 21.73, df = 3, P < 0.001) (**Table 1**). Across recovery media and sampling times, recovery was 40% in plants treated with *B. bassiana* + Silwet L-77, 26.3% with *B. bassiana* + Tween-20 and zero in controls. Both fungal treatments differed from the control (*B. bassiana* + Silwet L-77: Tukey-adjusted P = 0.001; *B. bassiana* + Tween 20: 0.007) whereas recovery did not differ between *B. bassiana* + Silwet L-77 and *B. bassiana* + Tween-20 (P = 0.106). Across seed treatments and sampling time, recovery was 27.5% on SDAY and 16.7% on PDA (P = 0.017).

**Table 1:** Summary of type II Wald χ² tests for a bias-reduced binomial generalized linear model predicting the probability of recovery of endophytic *Beauveria bassiana* from quinoa plants due to effects of block, seed treatment, recovery medium, and time.

Effect	df	χ²	*P*
Block	1	0.04	0.849
Seed treatment	2	15.17	<0.001
Recovery medium	1	5.68	0.017
Time	3	21.73	<0.001

Across seed treatments and recovery media, recovery was 38.3%, 31.7%, 16.7%, and 1.7% at 7, 14, 21, and 28 days after germination respectively. Recovery at 7 days was significantly higher than 21 and 28 days (Tukey-adjusted P = 0.02 and 0, respectively). Maximum recovery (70%) was observed at 7 days after germination in the *B. bassiana* + Silwet L-77 seed treatment with SDAY as recovery medium (**Figure 2**), and by 28 days after germination, *B. bassiana* was recovered only from plants in the *B. bassiana* + Silwet L-77 seed treatment with SDAY as recovery medium with 10% colonization.

**Figure 2:**
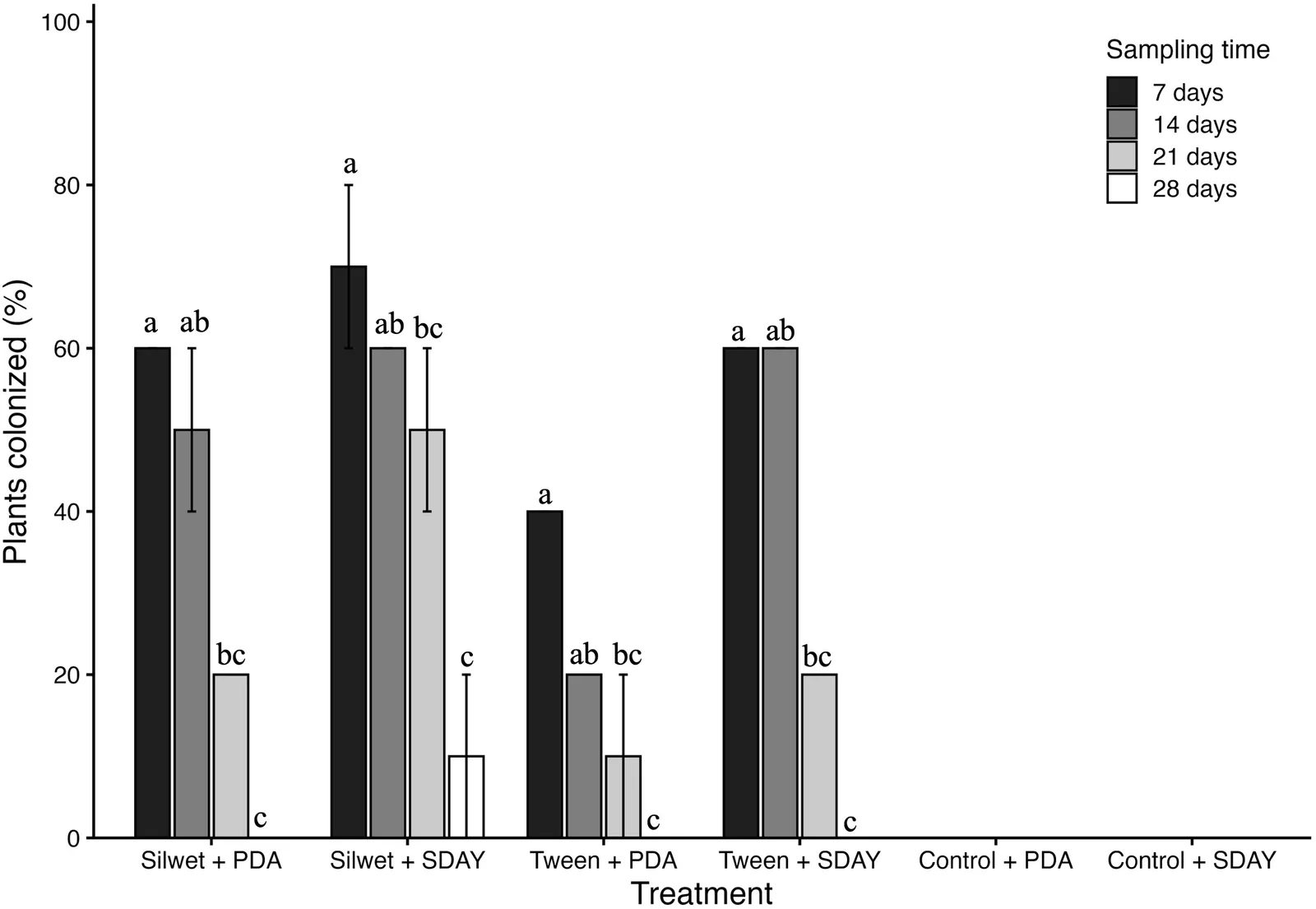
Endophytic colonization of quinoa by *Beauveria bassiana*. Bars show the percentage of plants colonized at 7, 14, 21, and 28 days after germination. For each treatment and time point, colonization percentage was calculated separately for each experimental block (five plants per block). Bars show the mean of the two blocks and error bars indicate the standard error of their mean. Treatments consisted of *B. bassiana* inoculated with Silwet L-77 or Tween, followed by fungal recovery on potato dextrose agar (PDA) or Sabouraud dextrose agar yeast extract (SDAY). Control seeds were soaked in sterile distilled water without *B. bassiana* or surfactant. Maximum colonization percentage was reported on treatment with *B. bassiana* + Silwet L-77 along with SDAY medium on all the sampling intervals. Different letters indicate significant differences among sampling times based on Tukey-adjusted pairwise comparisons of estimated marginal means averaged across surfactant and recovery-medium levels (P <0.05).

Block did not significantly affect colonization (χ^2^ = 0.04, df = 1, P = 0.849), and none of the interactions among block, seed treatment, medium, or time were significant (P > 0.1). Thus, data from both blocks were pooled for all analyses. None of our surface disinfection controls showed any contamination.

*Beauveria bassiana* was recovered from roots, stems, and leaves of seed-inoculated quinoa plants (**Table 2**). At 7 days after germination, recovery from stem tissues was greatest in *B. bassiana* + Silwet L-77 seed treatment with SDAY as recovery medium (60 ± 20%) and *B. bassiana* + Tween 20 and SDAY as recovery medium (60 ± 0%). Also, *B. bassiana* + Silwet L-77 treatment had the highest recovery of endophyte in leaf tissues using SDAY as recovery medium (40 ± 20%). The colonization remained detectable in *B. bassiana* + Silwet L-77 treatment (SDAY recovery medium) until 28 days whereas colonization declined to 0% in all other treatments by 28 days.

**Table 2:** Percentage of quinoa plants with *Beauveria bassiana* recovered from roots, stems, or leaves following seed treatments.

Seed treatment	Recovery medium	Days after germination	Root (%)	Stem (%)	Leaf (%)
*B. bassiana* + Silwet L-77	SDAY	7	50 ± 10	60 ± 20	40 ± 20
		14	40 ± 0	40 ± 20	40 ± 0
		21	40 ± 0	20 ± 0	30 ± 10
		28	10 ± 10	10 ± 10	10 ± 10
*B. bassiana* + Silwet L-77	PDA	7	50 ± 10	40 ± 20	20 ± 20
		14	30 ± 10	50 ± 10	30 ± 10
		21	10 ± 10	20 ± 0	20 ± 0
		28	0 ± 0	0 ± 0	0 ± 0
*B. bassiana* + Tween 20	SDAY	7	50 ± 10	60 ± 0	60 ± 0
		14	50 ± 10	60 ± 0	40 ± 20
		21	20 ± 0	20 ± 0	10 ± 10
		28	0 ± 0	0 ± 0	0 ± 0
*B. bassiana* + Tween 20	PDA	7	10 ± 10	20 ± 0	20 ± 0
		14	10 ± 10	20 ± 0	20 ± 0
		21	10 ± 10	0 ± 0	0 ± 0
		28	0 ± 0	0 ± 0	0 ± 0
Untreated control	SDAY	7	0 ± 0	0 ± 0	0 ± 0
		14	0 ± 0	0 ± 0	0 ± 0
		21	0 ± 0	0 ± 0	0 ± 0
		28	0 ± 0	0 ± 0	0 ± 0
Untreated control	PDA	7	0 ± 0	0 ± 0	0 ± 0
		14	0 ± 0	0 ± 0	0 ± 0
		21	0 ± 0	0 ± 0	0 ± 0
		28	0 ± 0	0 ± 0	0 ± 0

Recovery medium used for detection included Potato Dextrose Agar (PDA) and Sabouraud Dextrose Agar supplemented with 1% yeast agar (SDAY). Plants were sampled at 7, 14, 21, and 28 days after germination. Values are mean ± SEM of percentages calculated separately from two experimental blocks, with five plants per treatment per sampling time in each block. Tissue recovery was scored as present when the fungus was recovered from at least one section of that tissue.

### 3.2 Efficacy of *B. bassiana* against larval and exit hole densities of *A. karli* in potted quinoa plants

The probability of infestation incidence was consistently lower in *B. bassiana*-treated plants than in control plants in both years, although the difference was not statistically significant. In 2024, infestation incidence was 4.2% in treated plants compared with 12.0% in control plants (χ^2^ = 1.02, df = 1, P = 0.313); in 2025, incidence was 20.0% in treated plants as compared to 41.7% in control plants (χ^2^ = 1.8, df = 1, P = 0.18; **Figure 3A**).

**Figure 3:**
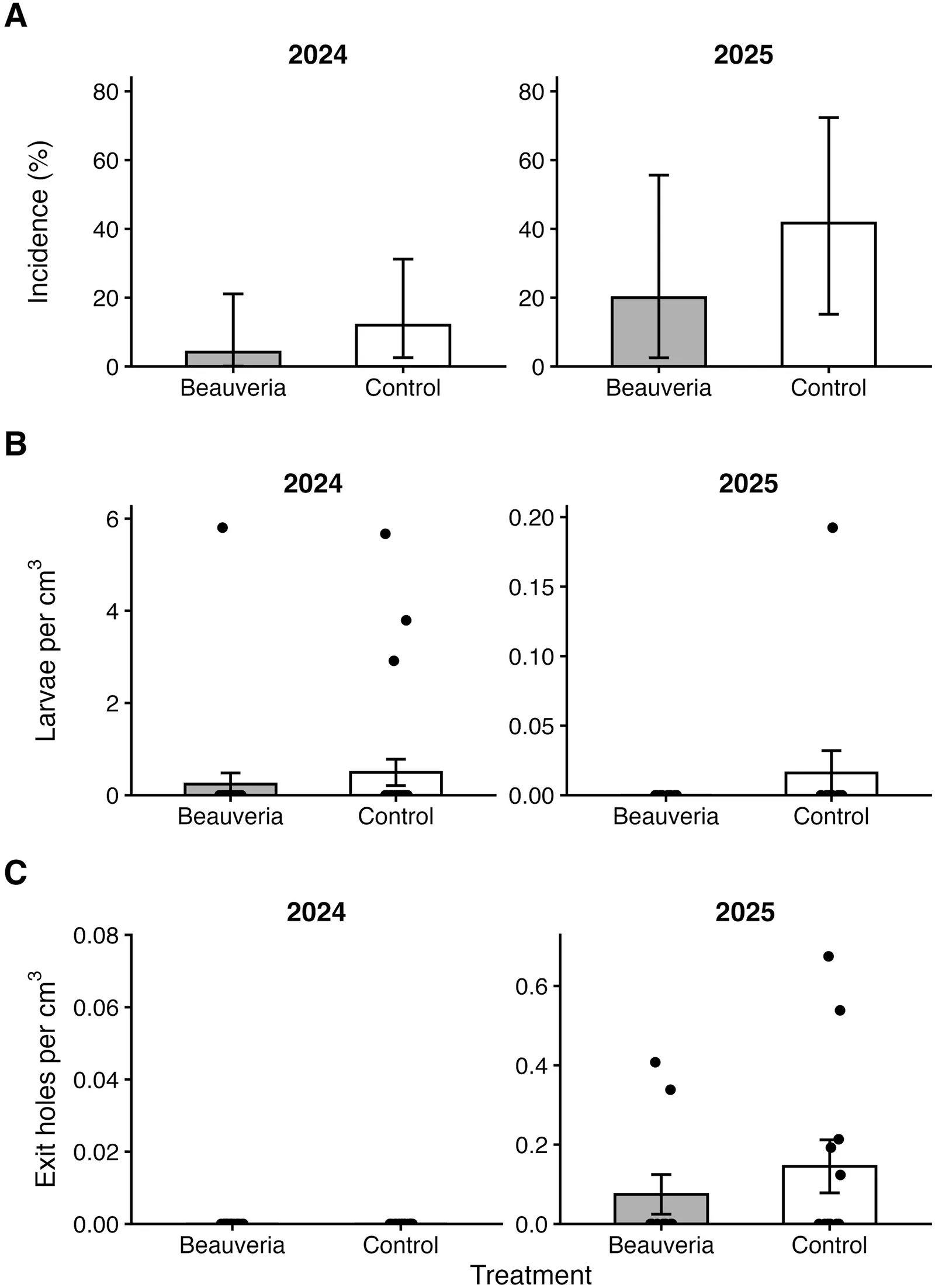
Effect of seed inoculation with *Beauveria bassiana* on infestation incidence **(A)**, density of larval *Amauromyza karli* **(B)**, and their exit holes **(C)** in potted quinoa following field exposure in 2024 and 2025. Treatments are on the x-axis, and bars represent treatment means ± SEM. Larval and exit hole densities are expressed per cm^3^ of plant tissue. No exit holes were observed in 2024.

Larval density did not differ significantly between treated and control plants in 2024 (F = 1.09; df = 1,44; P = 0.302; **Figure 3B**). Larval abundance was extremely low in 2025, with only one larva recovered across all sampled plants. No exit holes were observed in either treatment in 2024. In 2025, mean exit-hole density was approximately 49% lower in treated plants (0.075 ± 0.050 per cm^3^) than in control plants (0.145 ± 0.067 per cm³), however there was no significant difference (χ^2^ = 0.81, df = 1, P = 0.369; **Figure 3C**).

### 3.3 Efficacy of seed treatment with *B. bassiana* against *A. karli* infestation in directly sown field experiments

Seed inoculation with *B. bassiana* successfully established endophytic colonization in directly sown field plants, though this colonization was transient. At 14 days after sowing, mean endophytic colonization was 26.7 ± 8.4% in treated plants, whereas no colonization was detected in control plants. *Beauveria bassiana* was recovered from roots, stems, and leaves of 16.7 ± 6.1%, 20.0 ± 7.3%, and 10.0 ± 4.5% of plants, respectively. By the second sampling date, *B. bassiana* could no longer be isolated from either treated or control plants.

We found no significant difference among larval densities between treated and control plants (F = 0.18; df = 1,5; P = 0.686; **Figure 4A**). However, there was a significant difference among exit hole densities (F = 11.68; df = 1,5; P = 0.019; **Figure 4B**). Mean exit-hole density was 0.066 ± 0.022 exit holes/cm³ in the *B. bassiana* treatment compared with 0.165 ± 0.023 exit holes/cm³ in control plots, a reduction of approximately 59.7%.

**Figure 4:**
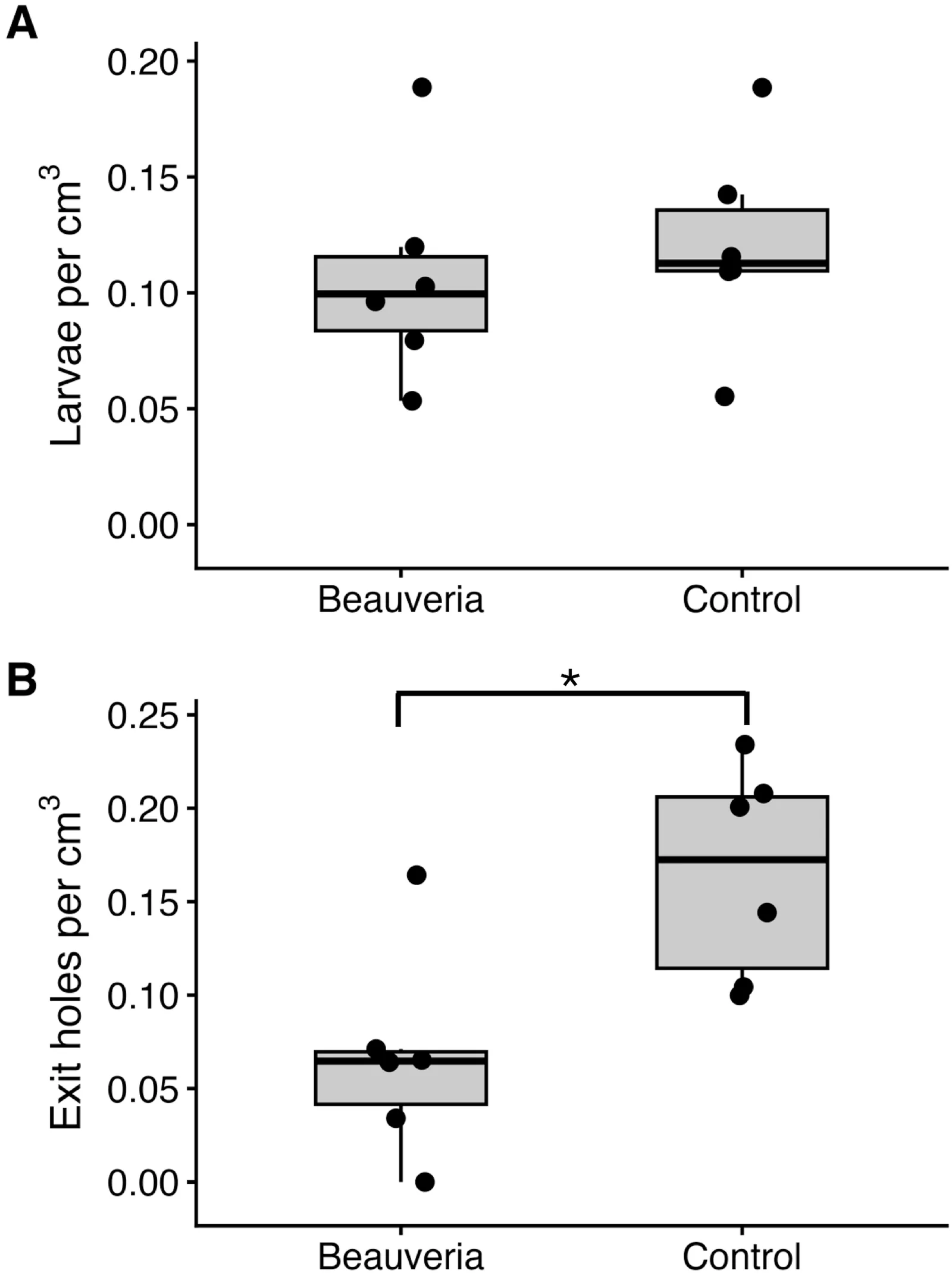
Box plot showing the effect of *Beauveria bassiana* on larval infestation **(A)** and exit hole formation **(B)** by *Amauromyza karli* in quinoa plots in 2026. Boxes represent the interquartile range, the horizontal line within boxes denotes the median, and individual points represent block means. Densities are expressed as the number per cm^3^ of stem tissue. Exit hole densities were lower in *B. bassiana* treatments than in control plants.

Stem length, stem diameter, and stem volume did not differ between treated and control plants. Mean stem length was 20.80 ± 0.98 cm in *B. bassiana-*inoculated plants and 21.09 ± 0.79 cm in control plants (F = 0.08; df = 1,5; P = 0.791). Mean stem diameter was 0.504 ± 0.010 cm and 0.497 ± 0.035 cm in treated and control plants, respectively (F = 0.04; df = 1,5; P = 0.848). Stem volume was 4.46 ± 0.32 cm^3^ in treated plants and 4.39 ± 0.54 cm^3^ in control plants (F = 0.02; df = 1,5; P = 0.906).

## 4 Discussion

To our knowledge, this is the first study to evaluate *B. bassiana* seed treatment against *A. karli* and to demonstrate reduced exit-hole density under field conditions. *Beauveria bassiana* have been previously shown to colonize only quinoa roots endophytically following soil inoculation (Alquichire- Rojas et al., 2024). In our study, we used seed treatment approach to introduce *B. bassiana* in quinoa plants and were able to recover it from belowground roots as well as aboveground tissues including stems, and leaves. We also found that inoculation of quinoa seeds with *B. bassiana* led to colonization of quinoa plants for up to four weeks after germination, with the highest frequency recorded seven days after germination and a decline at each subsequent sampling date. This transient pattern of endophytic colonization has also been reported in other studies. For example, Greenfield et al. (2016) reported that colonization of cassava by *B. bassiana* declined from 84% at 7–9 days post-inoculation to 40% at 47–49 days post-inoculation. Furthermore, Gurulingappa et al. (2010) found that colonization frequency by *B. bassiana* was lower at 21 days than at 7 days after inoculation, decreasing from 79% to 31% in cotton, 100% to 57% in wheat, and 89% to 27% in corn. Several mechanisms could account for this decline, including dilution of established fungal colonies as host tissue expands during growth, and competition with other endophytic fungi within the plant (Posada et al., 2007). Consistent with the latter mechanism, other fungal morphotypes were also observed emerging from surface-sterilized quinoa tissues during our recovery assays, suggesting that competition with other endophytes may have contributed to the decline in recovery of *B. bassiana* over time.

Although an economic injury level has not yet been established for *A. karli* in quinoa, field observations indicate that larval activity begins in early June and starts declining by mid-July (Panwar et al., 2026). Therefore, the relatively high recovery of *B. bassiana* during early plant development may coincide with the onset of *A. karli* infestation. However, because colonization declined over time, single seed-based inoculation may not maintain *B. bassiana* colonization through the entire quinoa development, but the period of greatest endophyte recovery may overlap with the early window of *A. karli* infestation. Supplementary inoculation strategies, such as foliar application or soil drenching at later growth stages, may extend the colonization window, although the effectiveness and timing of such applications need to be evaluated in future studies.

Despite the transient nature of endophytic colonization, seed inoculation with *B. bassiana* reduced density of exit holes. Exit hole density was reduced by 59% in directly seeded plants, whereas larval densities and plant growth parameters did not differ between treatments. Since exit holes are produced by mature larvae that exit the stem to pupate in soil, this suggests that *B. bassiana* did not prevent larval establishment or feeding within quinoa stems but may have either slowed larval development or disrupted the ability of larvae to complete development to the point of successful emergence. Similarly, Cherry et al. (2004) found that seed treatment with *B. bassiana* did not reduce larval infestation by *Sesamia calamistis* Hampson (Lepidoptera: Noctuidae) but lowered dead-heart formation by 27%. Furthermore, Zhang et al. (2023) found that larvae of potato tuberworm, *Phthorimaea operculella* (Zeller) (Lepidoptera: Gelechiidae) feeding on potato plants endophytically colonized by *B. bassiana* had a 52% reduction in pupal weight and only 17% developed to adult stages, indicating that effects may persist beyond the larval feeding stage.

No mycosed larvae were recovered from sampled plants in our study. Although the absence of external mycosis does not exclude the possibility of internal fungal infection, it suggests that direct larval mortality due to fungal infection was not common under field conditions. The lower exit-hole density indicates that fewer larvae completed development to the point of exiting stems during the observation period. Delayed development, plant-mediated responses, or fungal metabolites are hypotheses that warrant testing. However, these mechanisms were not directly evaluated in the present study and remains a hypothesis to be tested. Also, colonization and infestation were assessed in different individual plants, we cannot directly link endophytic colonization to lower exit hole densities. Future work is therefore needed to determine whether the observed suppression resulted from plant-mediated responses or indirect effects mediated by fungal metabolites or a combination of both, ideally by pairing colonization and infestation assessments on the same plants.

We observed different results in directly seeded quinoa and potted plants. This discrepancy may partly reflect differences in the timing and duration of exposure to *A. karli* activity. Directly seeded plants were exposed continuously to naturally occurring *A. karli* populations from emergence, when recovery of endophytic *B. bassiana* following seed inoculation was greatest. The potted plants were exposed to field populations for only 14 days in 2024 and 8 days in 2025, whereas directly seeded plants were continuously available for oviposition and larval establishment. This difference may be particularly important because approximately 50% of cumulative *A. karli* adult activity occurs by early July (Panwar et al., 2026), indicating that the potted plants entered the field after a substantial portion of seasonal adult activity had already occurred. Finally, the potted experiments involved smaller sample sizes than the directly sown experiment, which may have limited statistical power to detect an effect of similar magnitude to that observed in the field.

Recovery medium also influenced the detection of endophytic *B. bassiana*. The relative performance of different media depends on the host plant, tissue type, and fungal taxon. For instance, An et al. (2020) recovered a greater proportion of fungal endophytes on PDA than on SDAY from *Tupistra chinensis* (Asparagales: Asparagaceae). Similarly, Cheng et al. (2023) demonstrated that the largest number of endophytic fungi were isolated on PDA, although specific alternative media outperformed PDA for particular taxa. Selective media containing dodine is also commonly used for isolating *B. bassiana* because they suppress competing fungi while allowing growth of the target entomopathogen (Beilharz et al., 1982). These findings indicate that no single medium is optimal for isolating all fungal endophytes, and that medium composition can greatly affect the isolation of endophytes by differentially supporting fungal growth and the suppressing competitive microorganisms. In our study, SDAY performed better for recovering endophytic *Beauveria bassiana*. The additional peptone and yeast extract in SDAY as compared to PDA may have supported the emergence of *B. bassiana* from plant tissues containing low fungal biomass by suppressing saprophytic competitors (Ramírez-Rodríguez and Sánchez-Peña, 2016; Ramírez-Cáceres et al., 2026). Therefore, the greater recovery observed on SDAY may reflect the medium-specific differences in culturability and detection sensitivity rather than differences in actual colonization of the quinoa. These findings also indicate that estimates of endophytic colonization can be strongly influenced by the recovery medium used and that medium selection should be considered when comparing colonization frequencies among studies. For future studies, selective media specifically optimized for *B. bassiana*, including dodine-based media, may improve recovery of endophytic *B. bassiana* from plants. In addition, since culture-based detection can underestimate true colonization when fungal biomass is low, pairing with molecular detection assays such as species-specific quantitative PCR could provide a more sensitive and complete assessment of endophytic colonization (Garrido-Jurado et al., 2016).

In conclusion, our study underscores the potential of using endophytic entomopathogenic fungi to suppress *A. karli* in quinoa fields. However, several limitations of the experimental design should be considered when interpreting these results. Our efficacy experiments evaluated seed treatment formulations containing *B. bassiana* with either Silwet L-77 or Tween-20 relative to water alone. Because surfactant-only controls were not included, the observed results cannot be attributed specifically to *B. bassiana*. Therefore, differences in efficacy should be interpreted as a result of formulation containing both *B. bassiana* and surfactant rather than fungus or surfactant alone. Also, the arrangement of the potted-plant experiment and duration of field exposure differed between years, which limits direct comparisons of treatment effects across years. In addition, infestation depended on naturally occurring *A. karli* populations, and pest pressure was therefore not standardized among experiments or years. Finally, because yield was not measured, the observed reductions in infestations cannot yet be directly related to economic benefits. Overall, our findings suggest that *B. bassiana* can establish endophytically in quinoa and indicate that seed treatment with *B. bassiana* + Silwet L-77 may reduce infestation of *A. karli*. Future work should evaluate different fungal strains and inoculation methods to further improve the endophytic biocontrol of *A. karli*.

## Data Availability

The raw data supporting the conclusions of this article will be made available by the authors, without undue reservation.
